# Large modulation capacity in graphene-based slot modulators by enhanced hybrid plasmonic effects

**DOI:** 10.1038/s41598-018-34914-6

**Published:** 2018-11-15

**Authors:** Ran Hao, Ziwei Ye, YiJie Gu, Xiliang Peng, Hongsheng Chen, Erping Li

**Affiliations:** 1 Key Lab of Micro/Nano_Electronic Devices and Smart Systems, College of Information Science & Electronic Engineering, Zhejiang University, Hangzhou, 310027 China; 2 Zhejiang University-University of Illinois at Urbana-Champaign Institute, Zhejiang University, Haining, 314400 China

## Abstract

We present an effective scheme to improve the modulation capacity in graphene-based silicon modulator by employing the double slots configuration with hybrid plasmonic effects. Two modulators, i.e., metal-insulator-metal and insulator-metal-insulator configurations have been demonstrated, showing that the double slots design can significantly improve the modulation efficiency. The obtained modulation efficiency is up to 0.525 dB/μm per graphene layer, far exceeding previous studies. It can be found that the light-graphene interaction plays a pivotal role in the modulation efficiency, whereas the height of metal has profound influence on the modulation. Our results may promote various future modulation devices based on graphene.

## Introduction

Graphene optics, in tandem with silicon devices, have received much attention in the past two decades^1^. Among these devices, graphene-based optical modulators dictates sharply contrasting properties: the modulation speed, the bandwidth, and especially the efficiency can be improved by orders of magnitude, compared with silicon modulators^2^. In 2011, modulator composed of single-layer graphene on top of silicon waveguide was experimentally studied^3^, with an operation speed of 1.2 GHz, a broad optical bandwidth (1.35–1.6 μm), and a modulation efficiency (ME) of 0.1 dB/μm. In 2012, a modulator with two graphene layers was proposed^4^, working with improved modulation efficiency at 0.16 dB/μm. Further improvement has been demonstrated in a similar two-layer graphene based modulator in year 2013, with an improved modulation efficiency of 0.34 dB/μm^5^. A ultra-compact graphene-embedded silicon waveguide modulator was investigated with a high ME over 0.3 dB/μm^6^. In all these designs, the most difficult part is that the thickness of graphene is only 0.34 nm, but the corresponding optical wavelength in silicon is around hundreds of nanometers. Thus, there is a strong dimension mismatch between graphene and silicon, which indicates that graphene can only capture very few portions of the optical mode in these designs. Such drawback seriously limited the ME, e. g., the ME is only 0.1 dB/μm in ref.^3^. One possible solution we proposed before is to use the hybrid plasmonic effect in the slot waveguide to squeeze the optical field and enhance its integration with graphene^7^. However, in such design, the large portion of the mode leaks in the background. The potential of combining the versatility of graphene with subwavelength field confinement of plasmonic waveguides still need to be improved.

In this paper, we design novel modulators based on double-slots plasmonic waveguide (DSPW) with enhanced light-graphene interaction. Our results show that the ME in the designed DSPW modulators can be significantly improved than all previous results.

## Results and Discussion

According to different symmetries, two kinds of DSPWs have been proposed, i.e., the insulator-metal-insulator (IMI) configuration and the metal-insulator-metal (MIM) configuration

### Insulator-Metal-Insulator configuration

**Figure 1:**
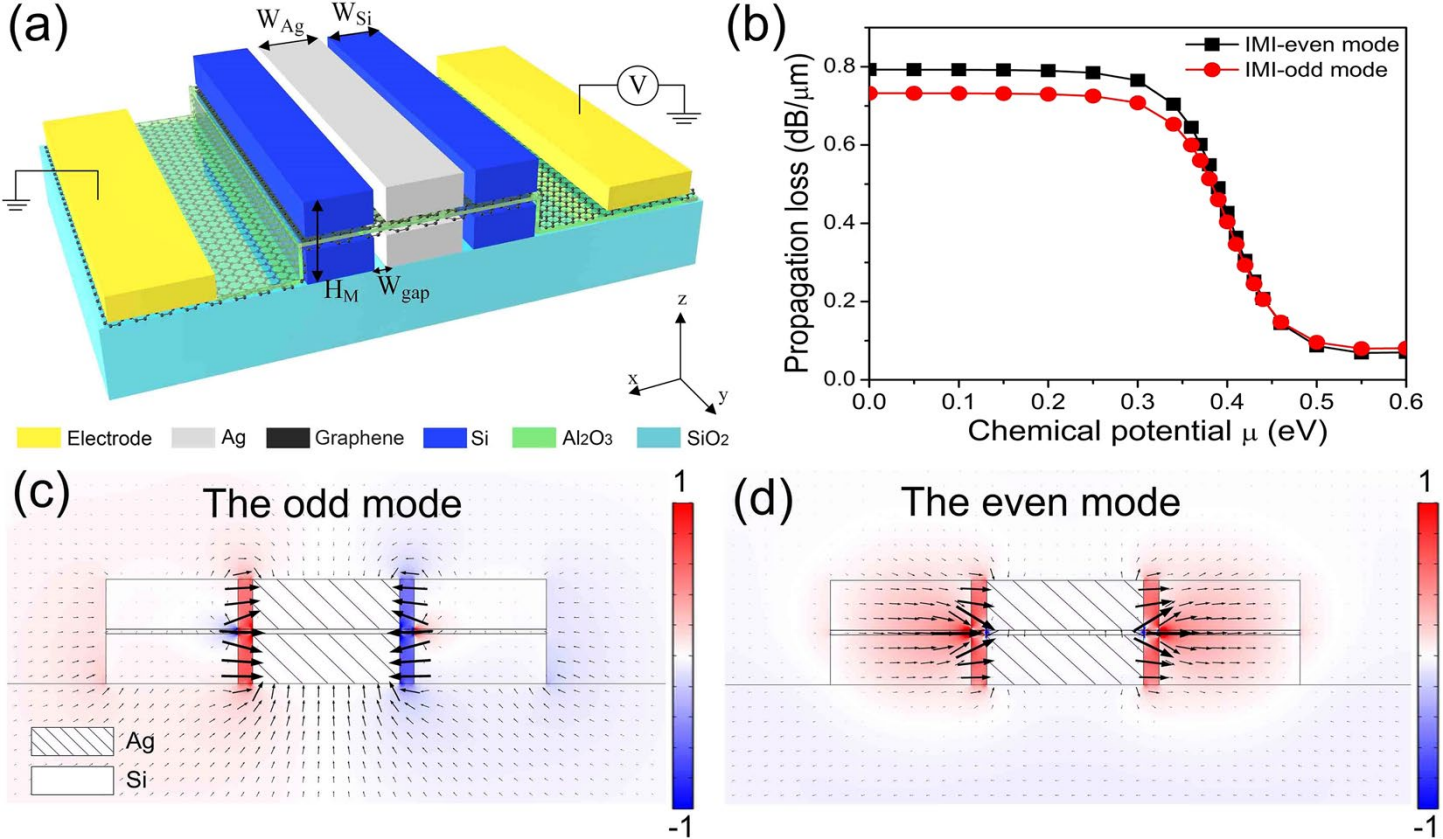
Schematic of the IMI-DSPW configuration; (**b**) Propagation loss of the IMI-odd and IMI-even mode under different chemical potentials; (**c**,**d**) Normalized electric field (*E*_*x*_) distribution for the IMI-odd (**c**) and IMI-even (**d**) mode.

The IMI configuration is illustrated in Fig. 1(a), where the silver (*n*_*Ag*_ = 0.1453 + 11.3587*i*) rib waveguide (*W*_*Ag*_ = 300 nm) is sandwiched between two silicon blocks (*n*_*Si*_ = 3.45, *W*_*Si*_ = 270 nm) to form a compound hybrid plasmonic waveguide (*H*_*M*_ = 220 nm, *W*_*gap*_ = 30 nm) on the surface of a *SiO*_*2*_ substrate (*nSiO*_*2*_ = 1.45). In this design, the double-layer graphene isolated by 10 nm *Al*_*2*_*O*_*3*_ layer (${n}_{A{l}_{2}{O}_{3}}$ = 1.746) have at least two functionalities. Firstly, they work as a capacitor, where the carriers between the two graphene layers form the required modulation. Secondly, graphene serves as a tunable absorber to absorb light dynamically. For an un-doping graphene, the carriers in the valence band can absorb the incident photons and jump to the conduct band^8^, thus the incident light suffers from deteriorated losses and attenuates. When the chemical potential is up to a certain value, the conduct band states are fulfilled, thus the incident photons cannot be absorbed and subsequently transmit in DSPW. The drastic change of propagation loss indicates that our graphene-based DSPW can configure modulator, where the propagation losses are manipulated by proper control of graphene’s chemical potential. The incident wavelength is *λ*_0_ = 1550 nm. In Fig. 1(b), the propagation losses for the intrinsic modes of DSPW are calculated from the imaginary part of the effective mode index as follows $\alpha =40\pi (\mathrm{lg}\,e)\text{Im}({n}_{eff})/\lambda {,}^{10}$ where a large variation in the propagation loss can be observed. High propagation loss (*μ* = 0 eV) can be set as “Off”, and low propagation loss (*μ* = 0.55 eV) can be set as “On” for a modulation.

In Fig. 1, the intrinsic mode is isolated by the central metal rib. The corresponding field from rigorous mode analysis displays a complex behavior: strong field concentration is observed in the two slots region, while weak leaking field is left in the background and less energy is detained inside the two silicon regions. According to the coupled mode theory^9^, there are two coupled modes in a pair of silicon waveguide named the odd symmetrical mode and the even symmetrical mode. In Fig. 1(c), the odd mode of IMI-DSPW displays an anti-symmetry where the signs of *E*_*x*_ fields at the two sides are opposite. In Fig. 1(d), the even mode of IMI-DSPW explicates a symmetric *E*_*x*_ field distribution where the signs of *E*_*x*_ fields at the two sides are the same. This also can be found from the arrows in Fig. 1(c,d) which represents the directions of the electric field.

**Figure 2:**
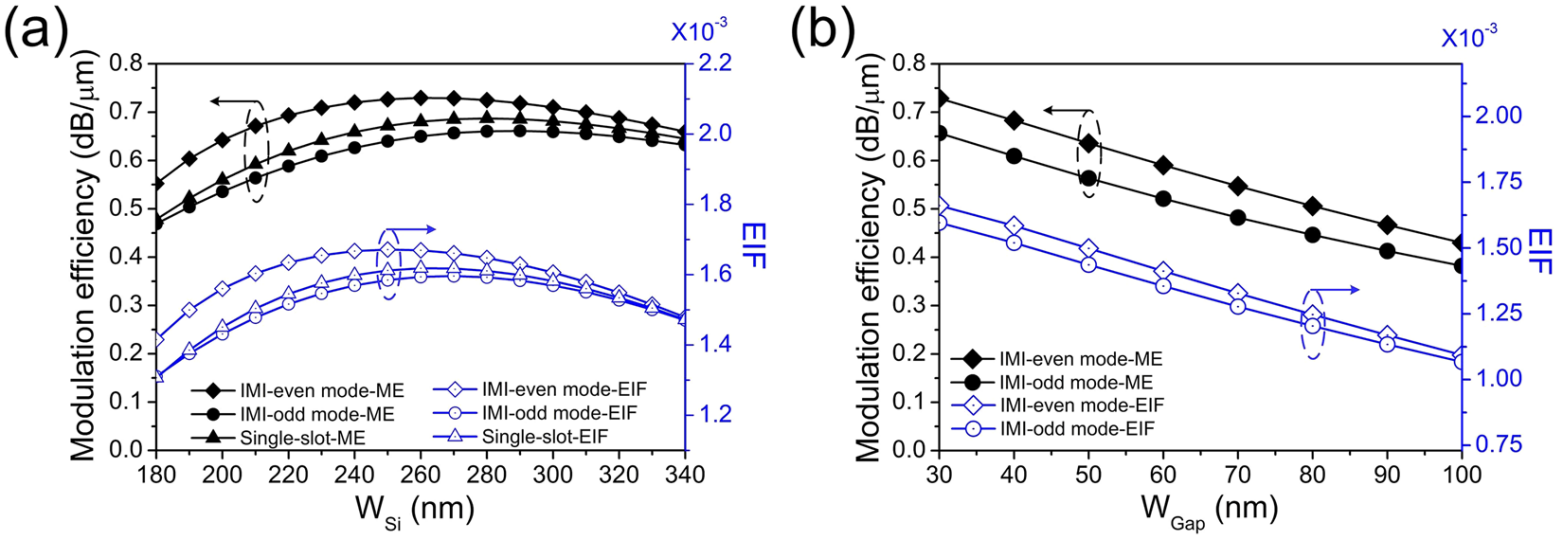
Modulation efficiency and effective interaction factor vary with different *W*_*si*_ (**a**) and under different W_*Gap*_ (**b**) for IMI-even mode modulator, odd mode modulator and single slot modulator.

In accordance with previous studies^10^, ME is used here to estimate the potential modulation capacity. ME is defined as the modulation depth (the ratio between output signal 1 and signal 0) per micro-meter. Figure 2(a) shows that the ME and effective interaction factor (EIF) varies when *W*_*Si*_ increases from 180 to 340 nm. Here, EIF is defined in the signal 0 state to quantitively study the graphene-light interaction:

1 $$EIF=\frac{{\int }_{G}{|{E}_{{\rm{p}}}|}^{2}dG}{{\int }_{S}{|{E}_{{\rm{P}}}|}^{2}+{|{E}_{\perp }|}^{2}dS}$$

where ${E}_{{\rm{P}}}=\sqrt{{{E}_{x}}^{2}+{{E}_{y}}^{2}}$ and ${E}_{\perp }=|{E}_{z}|$ are in-plane electric field intensity and out-of-plane electric field intensity separately. G is the area of the graphene region on the slot and S is the whole region^11^. EIF represents the degree of the light concentration in graphene, and the more the optical field concentrates in graphene, the more modulation capacity can be obtained. In Fig. 2, the ME behaviors are compared among the even mode of IMI-DSPW, the odd mode of IMI-DSPW, and single slot waveguide. In all three configurations, the ME curves have shown the similar trends: they all increase firstly and then decline when *W*_*Si*_ continuously increases. The even mode of IMI-DSPW has the highest ME for all the *W*_*Si*_ values because it has the strongest confinement. On the other hand, the odd mode of IMI-DSPW has the worst ME performance, because the odd mode of IMI has the smallest field concentrated in graphene according to smaller EIF as shown in Fig. 2(a). Furthermore, the slot width is critical for the modulation performance. As depicted in Fig. 2(b), the ME decreases with the increase of gap width. These EIF curves exhibit similar behavior with the ME curves, which suggests that EIF is an important indicator for ME.

### Metal-Insulator-Metal configuration

**Figure 3:**
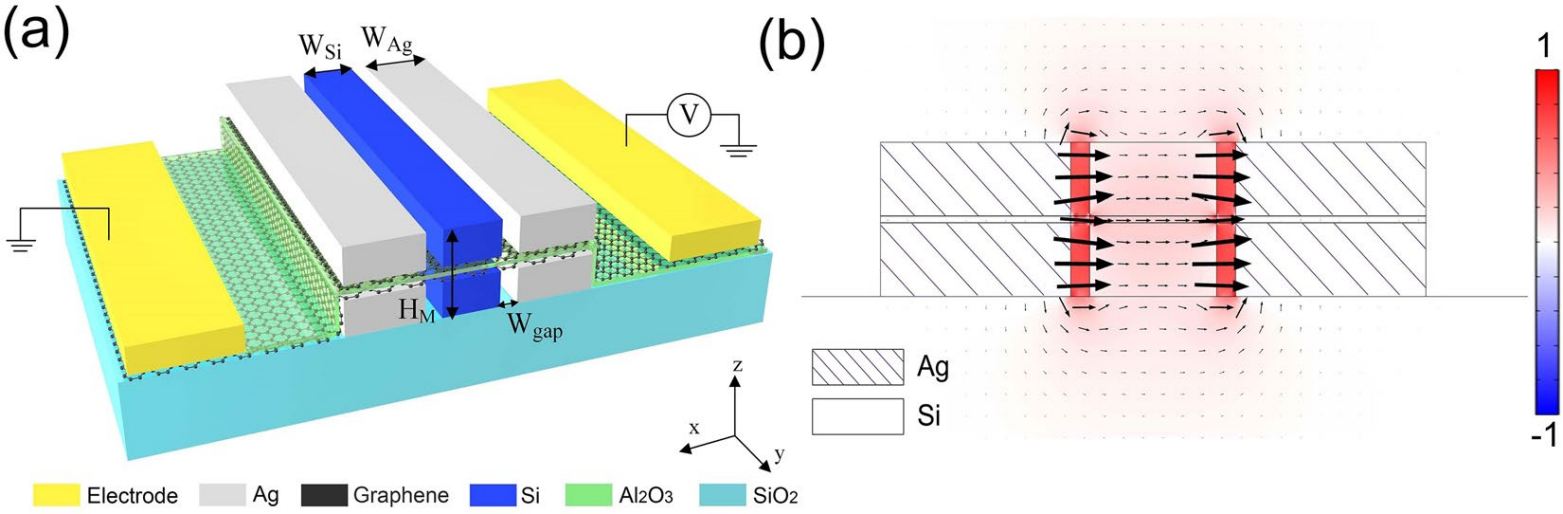
(**a**) Schematic illustration of the proposed MIM-DSPW structure. (**b**) Normalized electric field (*E*_*x*_) distribution of MIM-DSPW mode.

In Fig. 1(c,d), leaking fields are observed around the two sidewalls and the background, indicating low ME. These leaking fields are gathering because the dielectric waveguides are placed on the two sides. To avoid these side-wall leaking fields, another symmetry of the two slots configuration has been studied here: the metal-insulator-metal (MIM) configuration. The proposed configuration is depicted in Fig. 3(a), where two air slots (*W*_*Gap*_ = 30 nm) are inserted between the silicon rib (*W*_*Si*_ = 200 nm, *H*_*M*_ = 220 nm) and the two Ag waveguides (*W*_*Ag*_ = 300 nm). Two graphene layers are embedded in the compound waveguide with an isolated 10 nm *Al*_2_*O*_3_ layer. Figure 3(b) is the *E*_*x*_ field distribution for the designed MIM configuration. Unlike previous IMI case, the leaking modes gathering at the two sidewalls disappear, and all the intrinsic modes are concentrated in the central region, most of which are filled inside the slot areas. This indicates a much stronger EIF in the MIM structure than in the IMI structure, thus a larger ME shall be expected in the MIM structure.

**Figure 4:**
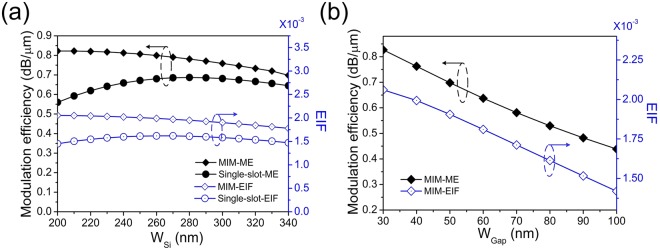
(**a**) Modulation efficiency and EIF of MIM-DSPW and single slot modulator under different *W*_*si*_. (**b**) Modulation efficiency and EIF of MIM-DSPW under different *W*_*Gap*_.

Figure 4(a) plots the ME performances for MIM configuration with different *W*_*Si*_. As a comparison, the ME for single slot waveguide is also plotted. The MIM configuration significantly improves ME with the maximum ME up to 0.82 dB/um, which is not only larger than the single slot waveguide but also larger than the largest ME value for the IMI-DSPW in Fig. 2(a). Furthermore, with the increase of *W*_*Si*_ from 200 nm to 340 nm, the ME for MIM-DSPW gradually decreases, because the increased central silicon region leads to a weaker light confinement. For the MIM configuration, it can be found that the ME/EIF is inversely proportional to the slot width, as can be seen in Fig. 4(b). The smallest *W*_*Gap*_ has the largest ME. In Fig. 4, it can be concluded that the EIF exhibits the same trend with ME, which suggests a convenient way to estimate the ME.

### Optimization

**Figure 5:**
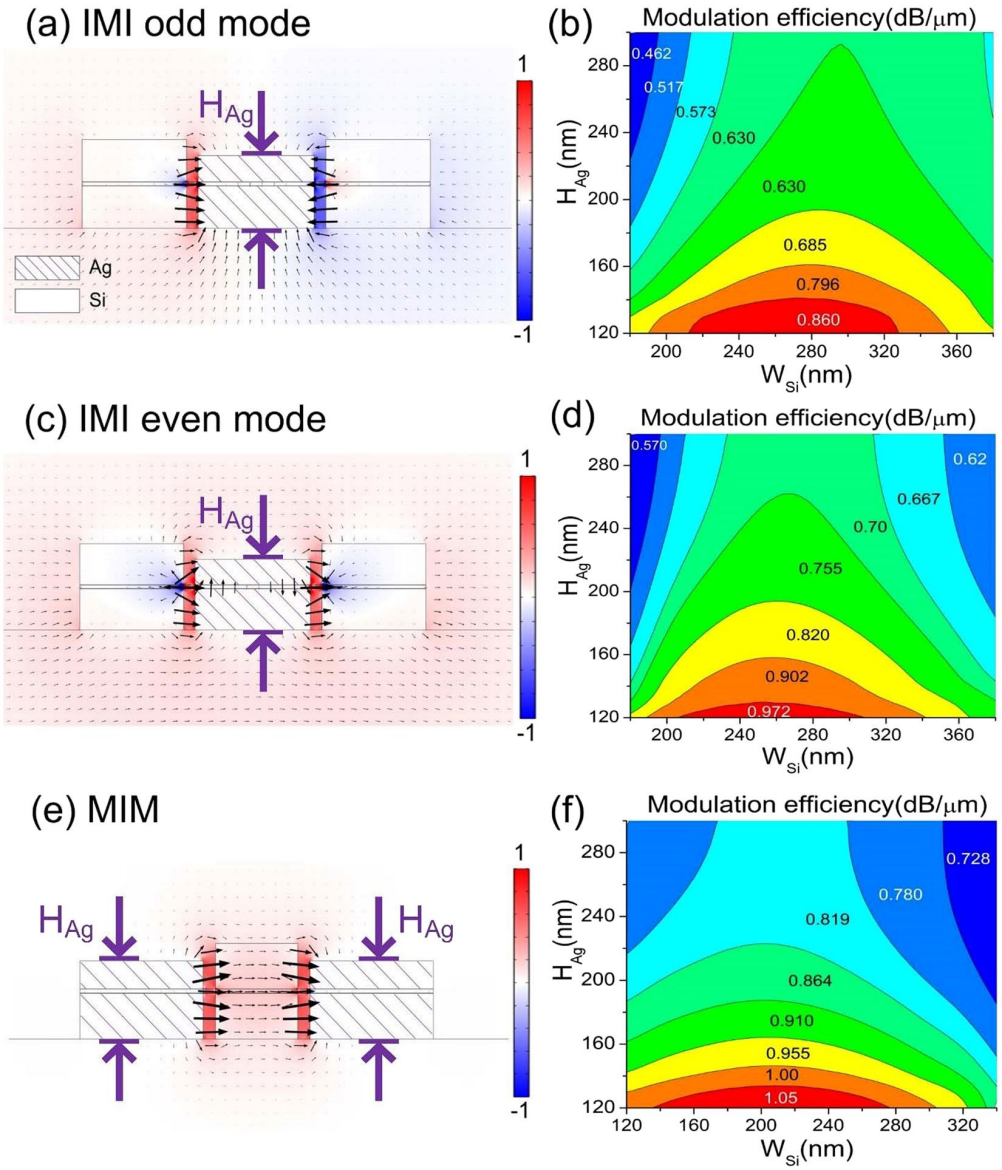
(**a**,**c**,**e**) Represents normalized electric field (*E*_*x*_) distribution of odd mode, even mode of IMI-DSPW and MIM-DSPW mode with 170 nm *H*_*Ag*_, respectively. (**b**,**d**,**f**) represents modulation efficiency of odd mode, even mode of IMI-DSPW and MIM-DSPW mode, respectively, under the different height of Ag waveguide *H*_*Ag*_ and width of the silicon waveguide *W*_*si*_.

According to previous plasmonic knowledge^12,13^, the surface plasmon is localized and significantly enhanced at the sharp corners of metal. Therefore, the idea here is to introduce the sharp corners of the metal inside the double-slots region. We deliberately reduce the height of the Ag region of the DSPW, as depicted in Fig. 5(a,c,e). Figure 5(a,c) are the schematic pictures of our design and the corresponding field distributions for the odd mode and even mode of the IMI-DSPW configuration; Fig. 5(e) is the schematic pictures of the improved design and the corresponding field distributions for MIM-DSPW configuration.

Systematic sweeps of the geometrical parameter variation for the modulation performances are performed, for the odd mode of IMI-DSPW in Fig. 5(b), the even mode of IMI-DSPW in Fig. 5(d), and MIM -DSPW in Fig. 5(f), respectively. Here we focus on two critical parameters: height of Ag waveguide *H*_*Ag*_ and width of the silicon waveguide *W*_*si*_. Since Ag is separated into two parts by the insertion of the graphene layer, only the top part of *H*_*Ag*_ is modified while the bottom part keeps constant at 105 nm. Our results show *H*_*Ag*_ has dominate influence on ME: the decrease of *H*_*Ag*_ significantly enlarges ME in Fig. 5(b,d,f). This is because the decrease of *H*_*Ag*_ not only introduces sharp metallic corners in the slot region, which enhances the hybrid plasmonic effects, but also enlarge the EIF factor according to Eq. (1). On the other side, *W*_*Si*_ has shown less influence but more complicated behavior on ME. For a fixed *H*_*Ag*_, with the increase of *W*_*Si*_, the ME first increases then declines. However, the peak value of ME gradually becomes larger *W*_*Si*_ with the increase of *H*_*Ag*_. For overall consideration, it can be concluded that the best ME performances for the odd mode of IMI-DSPW are 0.86 when *H*_*Ag*_ is between 120 nm and 140 nm and *W*_*Si*_ is between 210 nm to 330 nm; the best ME performances for the even mode of IMI-DSPW is 0.972 when *H*_*Ag*_ is between 120 nm and 130 nm and *W*_*Si*_ is between 210 nm to 310 nm; the best ME performances for MIM-DSPW is 1.05 when *H*_*Ag*_ is between 120 nm and 130 nm and *W*_*Si*_ is between 140 nm to 275 nm. It can be clearly seen that the MIM-DSPW exhibits the best efficiency for improving the ME.

### Comparison

There are plenty of designs on the topic of graphene-based modulators including single-layer graphene^3,14^, bi-layer graphene^4,15^, four-layer graphene^7^ and even eight-layer graphene^16^. All these studies reported improved ME, but the degree of improvement is quite different. To rigorously compare the modulation performances, we normalized ME by the number of the graphene layer, which is ME per layer (Δ = 0.34 nm), as shown in Table 1.

**Table 1:** Comparison of normalized Modulation efficiency for graphene modulator.

Reference/Year	Graphene-based structure	Modulation Efficiency per graphene layer (Δ = 0.34 nm) (dB/μm)
^3^/2011	Silicon waveguide	0.1
^17^/2016	Slot waveguide	0.144
^18^/2016	Hybrid plasmonic single slot waveguide	0.185
^7^/2017	Hybrid plasmonic single slot waveguide	0.21
^6^/2018	MZ modulator in electro-absorption modulation mode	0.3
Ours1/2018	IMI-DSPW	0.486
Ours2/2018	MIM-DSPW	0.525

In this paper, the double slots configuration has further improved the ME per layer. The ME per layer for even mode of IMI-DSPW has improved to 0.486 dB/μm, and for MIM-DSPW, it has improved to 0.525 dB/μm. To our knowledge, this is also the largest ME per graphene layer ever reported. This paper mainly focuses on the theoretical investigation and the optimization of the modulator performances. Although the fabrications of the proposed sandwich structures are quite challenged for the present clean room technology, the previous literature^12^ have shown the feasibility of experimental verification of graphene-based plasmonic modulators.

In conclusion, we propose a novel IMI-DSPW and MIM-DSPW modulator to significantly improve the modulation capacity. The modulation efficiency of the proposed MIM-DSPW is over ~0.525 dB/μm per graphene layer. The ME and corresponding EIF under different DSPW approaches and geometrical parameters are also investigated. It is found that the EIF is of great importance to the ME, and the height of metal also dramatically influences the modulation efficiency. Our results may benefit the design of high-performance on-chip electro-optical modulator.

## Methods

### Material modeling

Graphene is an anisotropic material, its permittivity in the out-of-plane direction ${\varepsilon }_{\perp }$ is a fixed at 2.5, while its in-plane permittivity ${\varepsilon }_{\parallel }$ can be expressed as:

2 $${\varepsilon }_{\parallel }=1+i{\sigma }_{g}/(\omega {\varepsilon }_{0}{\rm{\Delta }})$$

where Δ = 0.34 nm is the thickness of graphene^7^, and ${\sigma }_{g}$ is the complex conductivity of graphene:

3 $$\begin{array}{rcl}{\sigma }_{g}(\omega ,\mu ,{\rm{\Gamma }},{\rm{T}}) & = & {\sigma }_{\mathrm{int}ra}+{\sigma }_{\mathrm{int}er}=\frac{-i{e}^{2}}{\pi {\hslash }^{2}(\omega +i2{\rm{\Gamma }})}[{\int }_{0}^{\infty }\varepsilon (\frac{\partial {f}_{d}(\varepsilon )}{\partial \varepsilon }-\frac{\partial {f}_{d}(-\,\varepsilon )}{\partial \varepsilon })d\varepsilon ]\\  &  & \,+\,\frac{-i{e}^{2}(\omega +i2{\rm{\Gamma }})}{\pi {\hslash }^{2}}[{\int }_{0}^{\infty }\frac{{f}_{d}(\varepsilon )-{f}_{d}(-\,\varepsilon )}{{(\omega +i2{\rm{\Gamma }})}^{2}-4{(\frac{\varepsilon }{\hslash })}^{2}}d\varepsilon ]\end{array}$$

where ${f}_{d}(\varepsilon )$ is the Fermi function, *e*, T, $\hslash$ represent the electric charge, temperature and Plank constant respectively. *ω* is the angular frequency, Γ is the scattering rate, and *μ* is the chemical potential of graphene.

### Simulation methods

The EM wave simulation in this paper is based on mode analysis on the cross-section of the waveguide. The principle is to use the intrinsic mode distribution to analyze the wave vector (propagation constant) of the waveguide, as a result, the real part of the propagation constant indicates the phase performance, and the imaginary part epotomizes the loss information. The eigenvalue solver of commercial software COMSOL was used to find modes of the hybrid waveguide. The effective index and propagation distance were determined from the real and imaginary parts of the eigenvalue. The extremities of the calculation region were given scattering properties to mimic the necessary open boundary conditions. A convergence analysis was conducted to ensure that the real and imaginary parts of the effective indices varied within 1%.
